# Steric Interference of Adhesion Supports *In-Vitro* Chondrogenesis of Mesenchymal Stem Cells on Hydrogels for Cartilage Repair

**DOI:** 10.1038/srep12607

**Published:** 2015-09-28

**Authors:** Revital Goldshmid, Shlomit Cohen, Yonatan Shachaf, Ilana Kupershmit, Offra Sarig-Nadir, Dror Seliktar, Roni Wechsler

**Affiliations:** 1 The Faculty of Biomedical Engineering, Technion-Israel Institute of Technology, Haifa 32000, Israel; 2 Regentis Biomaterials Ltd., Or Akiva 30600, Israel

## Abstract

Recent studies suggest the presence of cell adhesion motifs found in structural proteins can inhibit chondrogenesis. In this context, the current study aims to determine if a polyethylene glycol (PEG)-modified fibrinogen matrix could support better chondrogenesis of human bone marrow mesenchymal stem cells (BM-MSC) based on steric interference of adhesion, when compared to a natural fibrin matrix. Hydrogels used as substrates for two-dimensional (2D) BM-MSC cultures under chondrogenic conditions were made from cross-linked PEG-fibrinogen (PF) and compared to thrombin-activated fibrin. Cell morphology, protein expression, DNA and sulfated proteoglycan (GAG) content were correlated to substrate properties such as stiffness and adhesiveness. Cell aggregation and chondrogenic markers, including collagen II and aggrecan, were observed on all PF substrates but not on fibrin. Shielding fibrinogen’s adhesion domains and increasing stiffness of the material are likely contributing factors that cause the BM-MSCs to display a more chondrogenic phenotype. One composition of PF corresponding to GelrinC™—a product cleared in the EU for cartilage repair—was found to be optimal for supporting chondrogenic differentiation of BM-MSC while minimizing hypertrophy (collagen X). These findings suggest that semi-synthetic biomaterials based on ECM proteins can be designed to favourably affect BM-MSC towards repair processes involving chondrogenesis.

Articular cartilage of the joints is an avascular tissue with a cell content of less than 5% that is known to lack self-regenerative capacity^1^. Current approaches aim to regenerate cartilage by either filling the defect with exogenous cartilage producing cells, i.e. chondrocytes or human bone marrow mesenchymal stem cells (BM-MSC), or by using osteo-chondral and chondral auto/allografts^2^,^3^. Cell based technologies requiring a two-step procedure are laborious, costly and involve inconvenience to the patient. Grafts offer immediate filling of the defect but integration is far from optimal and may not conform to the contours of the lesion. The development of microfracture (MFx) by Steadman^4^ revolutionized the field and MFx has now become the first-in-line treatment, especially for lesions less than 2 cm^2^,^5^. This procedure involves formation of small perforations in the sub-chondral bone leading to a “super clot” rich in BM-MSC and growth factors in the lesion site^4^,^5^. Fibrin, the main structural component of the blood clot, is a central player in this tissue regeneration process; however, instead of producing hyaline cartilage, MFx’s fibrin clot often leads to the formation of fibrocartilage and scar tissue repair.

Though functional on a short term basis, fibrocartilage is inferior to the native hyaline cartilage in many aspects and is often associated with poor clinical outcomes, especially in long-term prognoses^6^,^7^. In recent years, there has been an accelerated development of biomaterials to be used as MFx adjuvants or enhancers to stimulate hyaline-like cartilage repair^8^,^9^. These materials are intended to stabilize the clot, serve as scaffolds that actively recruit incoming BM-MSC, or stimulate MSC differentiation via inclusion of autologous fractions containing growth factors^10^,^11^. One assumption underlying these technologies is that the poor quality of the repair tissue in MFx is due to the limited number of stem cells in the defect area. Accordingly, biomaterials are designed to rapidly increase the availability of endogenous stem cells at the site of injury, much like the cell-based procedures which use exogenous cells for the same purpose^12^. However, clinical studies from the last decade suggest that cell-based approaches are no more effective than MFx^13^,^14^ and though some MFx adjuvants show promising short-term data, longer-term efficacy data is not available^15^,^16^. Thus, it is possible that the proper microenvironment, rather than the number of cells present in the defect, may be the critical factor in obtaining hyaline-like cartilage following MFx. Therefore, there is a clear need for biomaterials that can better provide microenvironmental cues for chondrogenic differentiation of endogenous BM-MSC^17^.

A recent clinical trial in cartilage repair also suggests that semi-synthetic biomaterials can have a therapeutic benefit when displacing a fibrin blood clot from the MFx defects in focal injuries^18^. Others have shown through *in vitro* studies that fibrin’s cell adhesion motifs can inhibit chondrogenesis, underscoring the limitations of the blood clot in MFx^19^. Two animal studies in which fibrin sealants were employed, have demonstrated impaired cartilage repair^20^,^21^. Taken together, these studies suggest a detrimental role of fibrin in cartilage repair, possibly through a suboptimal microenvironment presented to resident BM-MSCs. We set out to better understand the basis for fibrin's adverse role in cartilage repair at the cellular level by evaluating chondrogenesis of BM-MSCs through regulation of cell adhesion and matrix stiffness. Our experimental approach employs a semi-synthetic substrate matrix made from denatured fibrinogen and polyethylene glycol (PEG), onto which BM-MSCs are cultured. The modified fibrinogen is rendered biologically impaired by virtue of the chemical reaction that is used to create the cross-linked hydrogel matrix. Specifically, precursor molecules of PEG-diacrylate (PEG-DA) and denatured fibrinogen are conjugated by the Michael-type addition and cross-linked by free-polymer polymerization, creating a PEG network that sterically shields the fibrinogen backbone and proves higher degrees of cross-linking—both of which are proportional to the PEG-DA content^22^. A commercial form of this matrix, GelrinC, which is currently used in clinic for treating focal cartilage injuries, is applied as liquid filler, cured *in-situ* using long-wave ultraviolet light, and is resorbed over the course of several months. *In-vitro* as well as *in-vivo* evidence suggests that GelrinC is gradually resorbed through surface mediated erosion as it is replaced by hyaline-like cartilage tissue^23^,^24^. The hydrogel is impermeable to cells and thus interaction with cells is expected to occur exclusively on its surface as the material is eroded^23^,^25^. To further test the hypothesis that cell adhesion capacity of a given biomaterial can influence chondrogenesis in MFx, the current *in vitro* study aims to determine if this modified fibrinogen-based matrix can support cartilage differentiation of BM-MSC, when compared to a fibrin clot. Towards that aim, a cohort of PEG-fibrinogen (PF) hydrogels—containing different amounts of PEG and fibrinogen—was systematically screened for inducing cell aggregation concomitant with chondrogenic differentiation of BM-MSC, while using fibrin clots as a simplified model for the blood clot obtained by MFx.

## Materials and Methods

### Fibrinogen PEGylation and PF hydrogel preparation

PEGylation of fibrinogen was performed as previously described by Plotkin *et al.*^26^. Human fibrinogen (Tisseel, Baxter Biosurgery) was solubilized in phosphate-buffered saline (PBS) followed by addition of urea to 8 M (final concentration) for protein denaturation. The denatured fibrinogen solution in PBS + 8 M urea (PBS-Urea 8 M) was subsequently reduced using tris (2-carboxyethyl) phosphine hydrochloride (TECP-HCl, Sigma) at a molar ratio of 1.5:1 TCEP to fibrinogen cysteines. Poly(ethylene glycol) diacrylate (PEG-DA) powder (CML, The Netherlands) was dissolved (280 mg/ml) in PBS-Urea 8 M solution, and added to the reduced fibrinogen solution at a PEG to cysteine molar ratio of 3:1. Next, the mixture was reacted for 3 hrs at 25 °C. The reaction product was precipitated by addition of 4 volumes of acetone. The precipitant was re-dissolved in PBS-Urea 8 M and purified by tangential flow filtration against PBS. Fibrinogen concentration was determined using Kjeldahl nitrogen determination (BÜCHI, Switzerland) and the PEG to Fibrinogen molar ratio was calculated using previously described gravimetric technique^27^. PF hydrogels were made using PF solutions (350 μl, 7 mg/ml fibrinogen) containing 0.1% (w/v) photoinitiator (Irgacure 2959, Ciba). The solutions were illuminated in 24 wells polystyrene plate with UVA light (λ = 365 nm, I = 5 mW/cm^2^) for 5 minutes in order to initiate polymerization. Fibrin clots were made from human fibrinogen component of Tisseel Lyo Two-Component Fibrin Sealant (Baxter) by mixing 310 μl (7 mg/ml) fibrinogen solution in PBS with 40 μl thrombin solution (5 u/ml) and incubating at 37 °C for 20 min. PF hydrogels as well as fibrin clots were washed twice with PBS (2 × 10 minutes at 37 °C) prior to cell seeding (n = 4 for each time point).

### Rheological characterization of hydrogels

The storage shear modulus (G′) of PEG-Fibrinogen hydrogel formulations and thrombin cross linked fibrin clots were measured on a AR-G2 parallel plate rheometer (TA instruments, New Castle, DE) equipped with a 20 mm diameter parallel plate geometry and a UV curing cell. Time-sweep measurements were performed on all samples using an angular frequency of 3 rad/s and 2% strain. For each PF composition, 200 μl of hydrogel precursor solution (n = 3) was loaded and cross-linked using UVA (λ = 365 nm, I = 5 mW/cm^2^) from an adjacent light source (IlluminOss Medical Inc). For fibrin cross-linking, a peltier plate geometry was set on 37 °C in order to form the clot *in situ* during the time-sweep measurement. The compositions of the various formulations as well as the (G′) values are shown in Table 1.

### BM-MSC cell cultures

Human bone marrow mesenchymal stem cells (BM-MSCs, Lonza) were purchased and expanded in MSCGM medium (Lonza) according to the manufacturer’s recommendations. BM-MSCs were sub-cultured using trypsin-EDTA (Lonza) and used at 5^th^ passage, unless noted otherwise. For induction of chondrogenic differentiation in 2D, BM-MSCs (2 × 10^4^ cells) were seeded onto freshly prepared PF substrates or fibrin clots (0.3 ml) in 24 well optic plates (EBD). The cell density during seeding was kept at 20,000 cells/cm^2^. The cell cultures were incubated in differentiation medium (high-glucose DMEM supplemented with 10 ng/ml TGF-β3, 10^−7^ M dexamethasone, 50 μg/ml ascorbate-2-phosphate, 40 μg/ml proline, 100 μg/ml pyruvate and 50 mg/ml ITS premix consisting of 6.25 μg/ml insulin, 6.25 μg/ml transferrin, 6.25 ng/ml selenious acid, 1.25 mg/ml bovine serum albumin (BSA) and 5.35 mg/ml linoleic acid). The cultures were maintained in differentiation medium for 1, 4, 7 or 10 days with medium replacements every 3-4 days.

### Viability of BM-MSC following chondrogenic differentiation

BM-MSC viability was determined on day 10 by calcein/ethidium live/dead assay. Briefly, the medium was removed and adherent cells were incubated in 1 ml phosphate buffered saline (PBS) solution containing 2 μM calcein acetoxymethyl ester and 4 μM ethidium homodimer-1 (Sigma-Aldrich) for 40 minutes on an orbital shaker at 37 °C with 5% CO_2_. After staining, the cells were washed twice with PBS (2 × 10 min) and microscopically imaged on an inverted fluorescence microscope (Nikon Eclipse TS100, Nikon, Japan) using a digital camera (Digital Sight, Nikon, Japan) and Nikon Nis-Elements F3.00 software (Nikon, Japan).

### Characterization of cell aggregation

In order to quantify the aggregation of cell cultures on the various substrates, BM-MSCs were stained for f-actin organization and morphometricaly analyzed. Briefly, cells on substrates were fixed in 4% formaldehyde for 1 hour and permeabilized with 0.3% TritonX-100 in PBS for 10 minutes. Cells were then washed twice with PBS and stained for F-actin using phalloidin-TRITC (sigma) diluted 1:800 according to manufacturer recommendation. Samples were washed three more times with PBS under gentle agitation (for 1 hr at 37 °C), then incubated with PBS overnight at 4 °C. The stained samples were scanned and analyzed using an automated confocal microscope (InCell Analyzer 2000, GE, USA). For each sample, 12 randomly selected fields were imaged with a 10x objective using autofocus software and a Cy3 filter set. The images were segmented using a multi scale top-hat transform based algorithm. The total surface area of the cell aggregates was calculated from the segmented images using InCell Analyser software (4.5–11440 version).

### Cell invasion measurements

Fibrin clots and PF hydrogels were labelled using N-hydroxy-succinamide-fluorescein isothiocyanate (NHS-FITC) (Thermo Scientific). The labelling was performed by soaking the hydrogels in PBS containing 0.05 mg/ml NHS-FITC overnight at room temperature. Excess dye was removed by extensive washing in PBS. BM-MSCs were seeded onto labelled materials at a density of 20,000 cells per gel and cultured for 7 days in chondrogenic medium. On day 7, cells were fixed, permeabilized and stained with TRITC-labelled phalloidin, as described above. Cells were observed for their morphology and penetration into the various materials with a Zeiss LSM-700 confocal microscope (Carl Zeiss, Oberkochen, Germany) using 572 nm and 520 nm filter sets for TRITC-phalloidin (F-actin) and FITC (hydrogels), respectively. Z-sections of the constructs were obtained with a 2.5 μm interval and a depth of 400 μm. Z-stacks were reconstructed into 3D images (both fluorescent channels) using Imaris software (Version 7.7.0, Bitplane, Switzerland). Image analysis using a Surface modulus algorithm was performed to measure the penetration depth of the cells. TRITC-phalloidin and FITC were segmented using a region-growing algorithm with a threshold voxel of 20 for a single cell. Cells on hydrogel surfaces were segmented separately from cells that penetrated into the hydrogel. The average distance between the two segmented cell populations was calculated using 40 different regions representing the most penetrating cells in the sample.

### Cell retention experiments

PF hydrogels or fibrin clots (0.1 ml, n = 4) were freshly prepared in 96-well plates and washed twice with PBS (2 × 10 minutes at 37 °C) prior to cell seeding. BM-MSCs cultured in Chondrogenic medium were seeded onto the prepared gels at a density of 40,000 cells/cm^2^ and incubated at 37 °C and 5% CO_2_ for the designated time points of the experiments. Unattached cells were carefully removed without compromising the integrity of the gels. Subsequently, each well was filled with 0.15 ml of chondrogenic medium. Alamar blue dye (10%) (AbDSerotec) was added to all wells including control wells containing hydrogels without cells for a period of 1–5 hours. Reduction of the dye was assessed by measuring the absorbance at 540 nm and 630 nm. The percentage of dye reduction was calculated according to manufacturer’s protocol.

### Immuno-staining for chondrogenic markers

Chondrogenic differentiation of BM-MSC samples harvested at day 7 was documented using immunohistochemistry. Briefly, cells on the hydrogels were fixed, permeabilized as described above and stained with mouse anti-collagen type-I (1:2000 dilution, Abcam), rabbit collagen type-II antibodies (1:200 dilution, Abcam), rat anti-collagen type-X (1:200 dilution, Abcam), or mouse anti-human Aggrecan (1:20 dilution, R&D). Parallel cultures were stained with proper isotype control antibodies. Primary antibodies were detected using both goat anti-rabbit CY2 (1:100 dilution, Jackson) and donkey anti- mouse 647 (1:200 dilution, Life Technologies). Plates containing the samples were gently agitated for 1 hour and TRITC-phalloidin (1:800 dilution, Sigma) was added to each sample. After 30 minutes incubation at room temperature, DAPI reagent (diluted 1:400, Sigma) was added to each sample and samples were incubated for an additional 1 hr at room temperature. Samples were then washed 3 × 10 minutes with PBS at room temperature and a final wash in PBS was performed overnight at 4 °C. Confocal microscopy and image analysis of chondrogenic markers Immuno-stained BM-MSC samples were selected randomly for imaging on a Zeiss LSM 700 confocal microscope. Two independent samples from each treatment were tested with twenty different regions per sample (Z-stack images were captured at a resolution of 1024 by 1024 pixels, using a 10X objective, a 2.5 μm interval depth and a total depth of 400 μm). Isotope control samples were used for subtracting the background fluorescence levels. Segmentation by Imaris software was performed for each marker, with the fluorescence intensity of each voxel measured and the intensity for the whole 3D reconstructed image expressed as the sum of the intensity at the different Z-stack levels. For each marker, the intensity sum value was normalized to that obtained for actin for the same region.

### Biochemical analysis for DNA and sulfated glycosaminoglycan

Fibrin and PF hydrogels (1.5 ml) were prepared as described earlier in 12 well plates. BM-MSC were seeded at a density of 20,000 cells/cm^2^ and grown for 7 days in a chondrogenic medium. Cells were harvested with trypsin/EDTA and lysed with 100 μl of papain extraction buffer containing 0.2 M sodium phosphate buffer (pH 6.4), 10 mM EDTA, 0.1 M sodium acetate, 7 mM L-cysteine and 0.12 mg/ml papain from papaya latex (Sigma-Aldrich) at 65 °C for 3 hours. DNA content was determined using 0.2 mg/ml Hoechst 33258 dye (Sigma-Aldrich) and quantified against salmon testes DNA standard. Samples were quantified with a fluorescence plate reader at 360/460 nm. GAG content was measured using dimethyl-methylene blue (DMMB) dye binding method^28^. Briefly, 50 μl of papain-digested sample were combined with 100 μl DMMB reagent solution (40 mM NaCl, 40 mM glycine, 46 mM DMMB, pH 3.0). The absorbance was determined at 525 nm using shark cartilage chondroitin sulfate C standard (Sigma-Aldrich).

### Statistical Analysis

Statistical analysis was done using the Microsoft Excel 2010 software. All the results were expressed as mean ± standard deviation (SD). Statistical significance was determined using Two-sample unequal variance, two-tailed distribution Student’s T-test. A value p < 0.05 was considered statistically significant. Analysis of correlation between material stiffness and cell aggregation was performed using a correlation coefficient as determined at day 7 using the CORREL function (Microsoft Excel 2010). The equation for the correlation coefficient is:


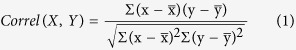


where 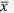 *and* 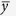
are the sample means.

## Results

### BM-MSC morphogenesis

**Figure 1:**
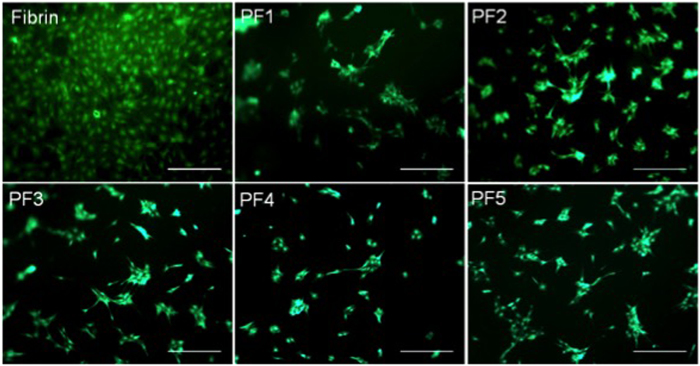
Viability of 2D BM-MSC cultures on fibrin and PF formulations. A qualitative staining assay for live and dead cells using calceinAM (live in green) and ethidium bromide (dead in red), was applied to all treatments. High viability was observed for each tested formulation at 10 days post seeding. Scale bar = 500 μm.

**Figure 2:**
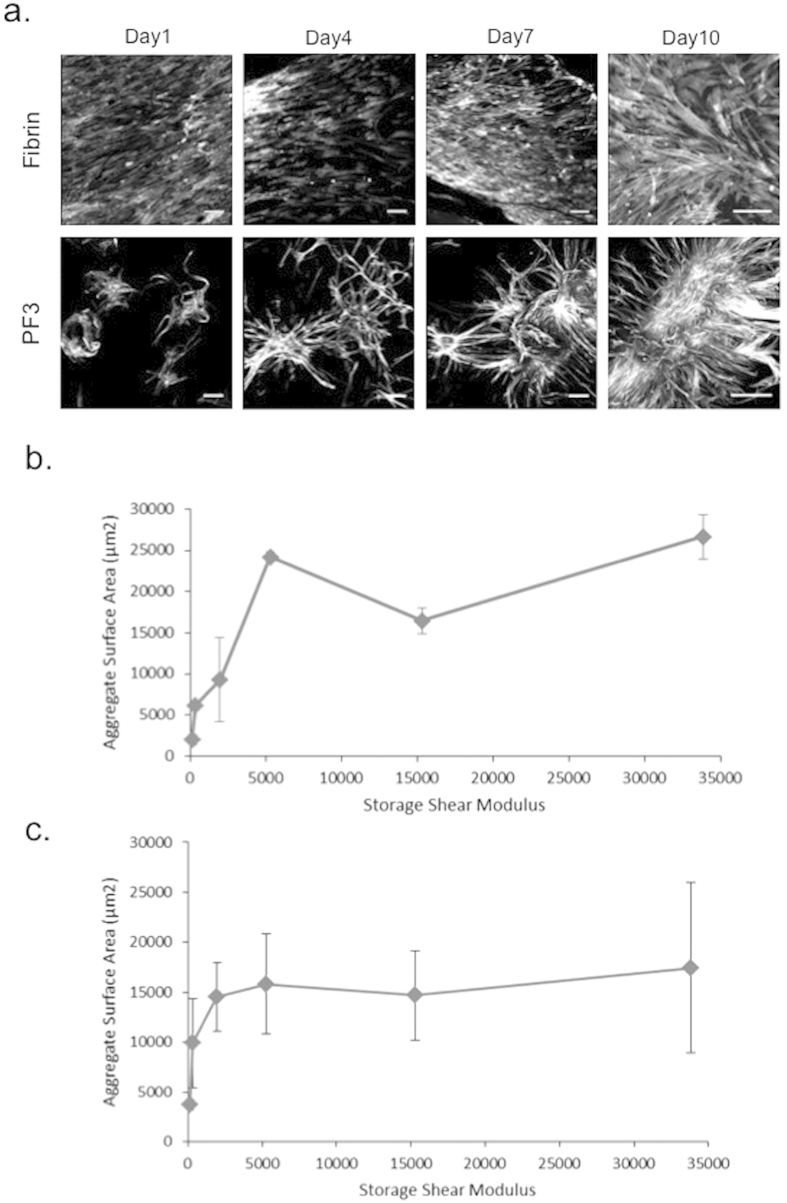
Cell morphology of 2D BM-MSC cultures on fibrin and PF formulations. (**a**) Images of 2D BM-MSC cultures seeded for 1, 4, 7 and 10 days on either fibrin or PF3 in chondrogenic medium and stained for f-actin; scale bar = 150 μm. (**b**,**c**) Quantification of aggregate area (in μm^2^) using an InCell Analyzer 2000. Aggregate area is plotted against storage shear modulus (G′) of the substrate on which the cells are cultured after 7 days (**b**) and 10 days (**c**) in culture.

Fibrin clots and different compositions of PF hydrogels, all made with 7 mg/ml fibrinogen, were characterized in terms of their shear storage modulus (Table 1). When BM-MSCs were cultured on these materials, their viability was found to be >90% throughout the culture period, irrespective of substrate type or modulus values (Fig. 1). The BM-MCSs formed discrete cell aggregates of various sizes on the PF formulations, whereas cells seeded on fibrin formed highly organized monolayers. Representative f-actin stained micrographs of BM-MSC cultures on fibrin and on PF3 are shown in Fig. 2a. As early as 24 hrs post-seeding, the cells on PF3 aggregated to form clusters with extensive branching structures. In contrast, cells seeded onto fibrin exhibited characteristic polarized fibroblastic morphology. By day 4, BM-MSCs formed interconnected multicellular clusters on PF3 whereas BM-MSC on fibrin kept a fibroblastic morphology with more ordered orientation. By day 7, BM-MSC seeded onto PF3 substrate exhibited larger and denser nodules with long processes radiating from their centres. Conversely, cells seeded on fibrin formed a very ordered, polarized sheet-like structure with distinct single-cell morphologies. Quantitative analysis of the average aggregate surface area was performed on PF samples in order to identify correlations between gel stiffness and extent of cell aggregation. Figure 2b summarizes the average aggregate area as a function of hydrogel modulus of each PF material. In general, there was a reasonable correlation across all formulations (correlation coefficient of 0.76).

### BM-MSC invasion and adhesion

**Figure 3:**
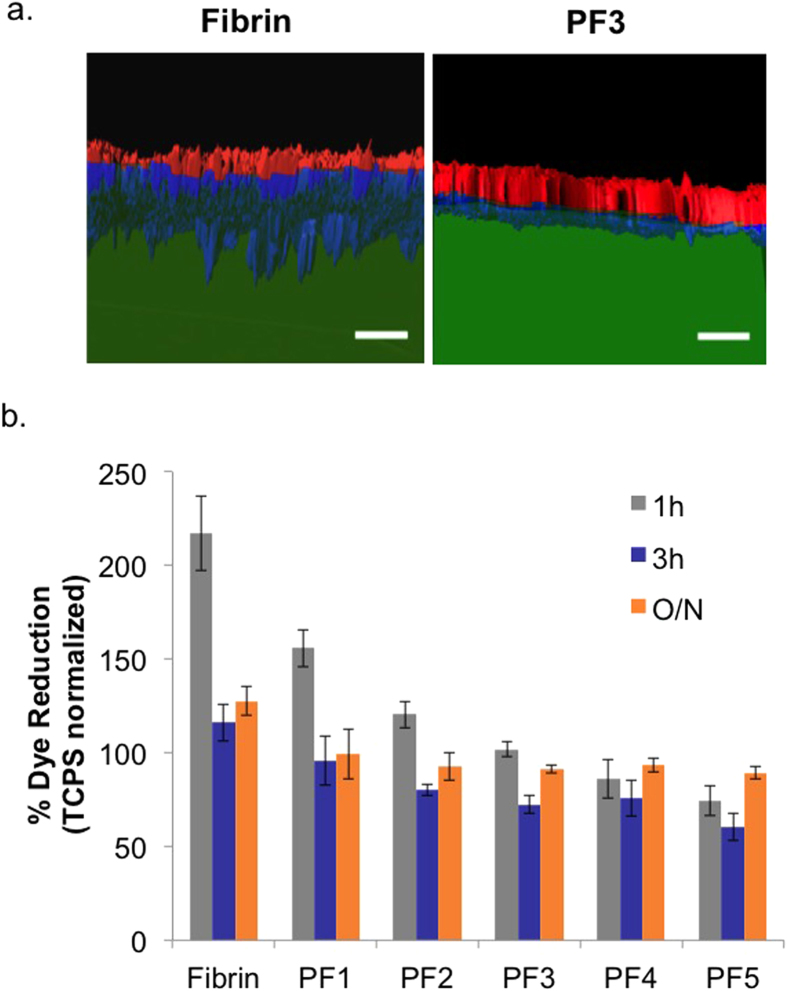
The interaction of BM-MSCs with fibrin and PF substrates. (**a**) Invasion of BM-MSC into fibrin or PF3 is shown with Imaris-segmented confocal images. The Z-stack 3D reconstructions of the cells stained for f-actin that were cultured on FITC-fibrin or FITC-PF for 7 days in chondrogenic medium. The cells located on top of the substrate are shown in red while those that penetrated the substrate material are shown in blue (materials are shown in green); scale bar = 150 μm. (**b**) Short term adhesion of BM-MSCs to fibrin and PF was documented with an Alamar Blue assay. The % dye reduction, which is proportional to cell number normalized to TCPS values, is shown for 1, 3 hrs and overnight (O/N) time points; n = 5 for each substrate material. These values are based on an initial cell seeding density of 40,000 cells/cm^2^.

The permeability of the substrates to BM-MSCs invasion was evaluated by Imaris analysis of Z-stacks of rhodamine-labelled cells on either FITC-fibrin or FITC-PF3 substrates in chondrogenic medium after 7 days (Fig. 3a). The images of hydrogel substrates, shown in green, and cells stained for f-actin, shown in either red (outside of gel) or blue (inside of gel), revealed extensive penetration of cells into the fibrin and much less penetration into PF3. Quantitative analysis of penetration depth into both gels (number of cells: n = 52 and n = 43 for fibrin and PF3, respectively) showed that cells invaded a distance of 175 microns into the fibrin substrates and 50 microns into the PF3 after 7 days. The adhesiveness of the fibrin and PF substrates to BM-MSCs was also evaluated during the first 18 hrs of cell culture (Fig. 3b) by seeding a fixed number of cells on the substrates and evaluating the metabolic activity of cells retained on the substrates after washing at set time-intervals. Figure 3b shows the metabolic activity of the attached cells, expressed as % of dye reduction and normalized to tissue culture polystyrene (TCPS). After 1 hour, cells retained on fibrin showed more than double the metabolic activity of the cells on TCPS. Among the tested PF formulations, there was an inverse correlation of decreased metabolic activity to PEG content with PF1-2 having higher activity than TCPS (but lower than fibrin) and PF3-5 having equal or lower activity. The difference between these formulations was lower after 3 hours (post-seeding), although a similar profile can be seen. After overnight incubation (O/N), all the PF hydrogels similarly affected the metabolism of the retained cells, although all showed reduced cell activity as compared to fibrin. There was a statistical difference between fibrin and all the PF hydrogels for all time points (p ≤ 0.05).

### BM-MSC chondrogenesis on Fibrin and PF substrates

**Figure 4:**
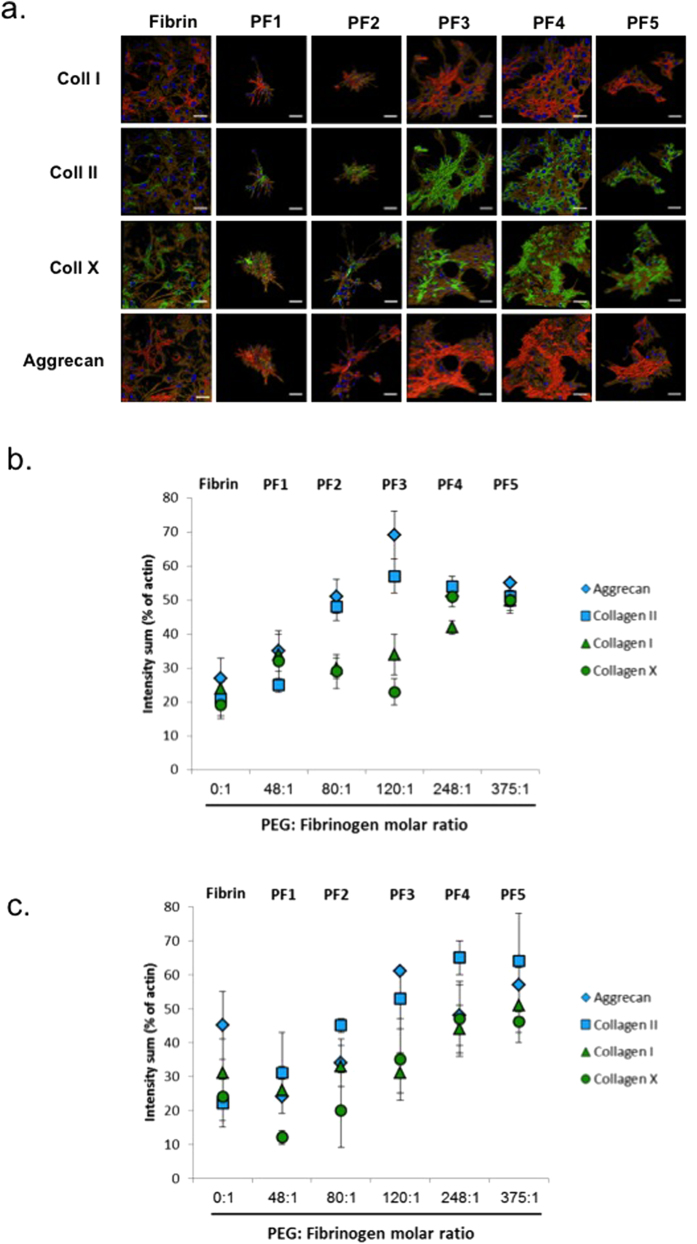
Chondrogenesis of BM-MSC in 2D culture. (**a**) Representative segmented confocal images of 2D BM-MSC cultures seeded onto fibrin and PF formulations for 7 days and immuno-stained for various chondrogenic protein markers; scale bar = 150 μm. (**b**,**c**) Quantification of immuno-staining for various protein markers using Imaris software at day 7 (**b**) and day 10 (**c**) in culture. For each tested material, two independent samples were tested with twenty different 3D regions per construct. The total fluorescent intensity of all regions for each marker (intensity sum) was normalized to that of actin and expressed as the normalized intensity (in %).

**Figure 5:**
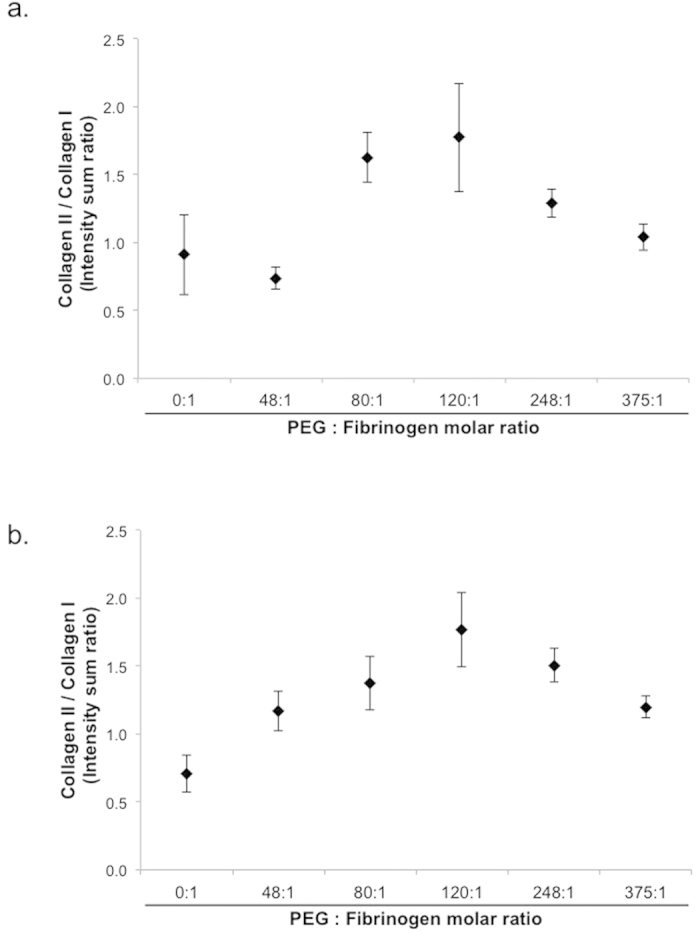
Ratio of collagen-II/collagen-I protein-level expression on different PF formulations. The ratios of intensity sum values for collagens-I and collagen-II are shown for day 7 (**a**) and day 10 (**b**) in culture. The molar ratio of PEG to fibrinogen for each formulation is shown.

**Figure 6:**
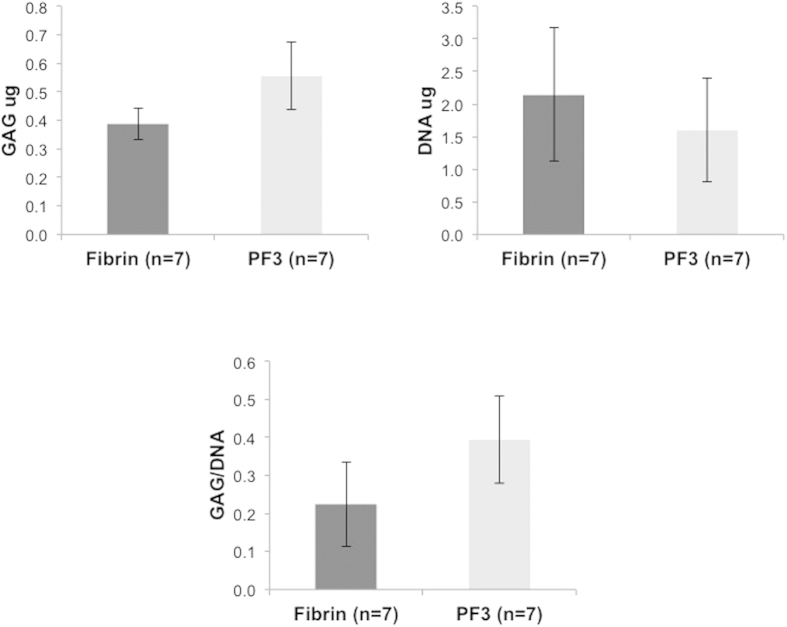
Biochemical analysis of GAG levels. Shown are DNA levels, total GAGs and normalized GAGs (GAGs/DNA) for BM-MSC cultured for 7 days on PF3 substrates (n = 7) and fibrin substrates (n = 7).

The chondrogenic differentiation of BM-MSCs on the different substrates was evaluated after 7 days in chondrogenic medium. Specific markers of hyaline cartilage, including collagens II and aggrecan, were imaged alongside a fibrocartilage marker, type-I collagen, and a hypertrophic cartilage marker, type-X collagen (Fig. 4a). Quantitative data from multiple images were obtained by segmentation using Imaris software (Fig. 4b). Representative segmented images of BM-MSC cultures seeded on each of the tested formulations shows a clear increase in staining intensity of collagen-II and aggrecan with increasing PEG content in PF hydrogels. A maximal staining intensity for both markers was found in cells seeded on PF3. Staining for collagen-I was variable, with cells seeded on fibrin and PF4-PF5 showing high staining, whereas those seeded onto PF3 exhibited the lowest values. Collagen-X was found to be lowest in cells seeded on PF and highest in PF4-PF5 gels (Fig. 4b). The ratio of collagen-II to collagen-I at the protein-level was determined using the staining intensity for each group (Fig. 5). The relative PEG content in the PF hydrogel groups proved consequential on this ratio, with a non-linear relationship apparent. The collagen-II/collagen-I ratio initially correlated to an increase in PEG:fibrinogen molar ratio, up to values of of 120:1 (i.e., PF3). Beyond that, any additional PEG was found to decrease the ratio of collagen-II/collagen-I. In order to further substantiate these findings, BM-MSCs were seeded on fibrin or PF3 for 7 days in chondrogenic medium, and biochemically analyzed for sulfated GAGs normalized to DNA content (Fig. 6). Cells seeded on PF3 had significantly more GAGs that those seeded on fibrin and almost a two-fold increase in DNA-normalized GAG levels (p < 0.05, n = 7).

## Discussion

The current *in-vitro* study investigated two types of biomaterials, a reconstituted fibrin clot and a semi-synthetic PF hydrogel, for their ability to support chondrogenic differentiation of BM-MSCs. Fibrin is often considered a suboptimal substrate of chondrogenesis, and its use in cartilage regeneration has been limited as a result. Moreover, fibrin-based blood clot, which is formed during the most common procedure for cartilage repair (microfracture), results in inferior fibrocartilage. The main novelty of the present investigation is the discovery that simple polymer modification of fibrin transforms this material into a matrix that is much more conducive to chondrogensis of BM-MSCs. The simple PEG modification to fibrinogen presented herein appears to substantially improve the material for chondrogenesis based on the influence that it has on the adhesion of BM-MSCs.

In this study, the concentration of fibrinogen in the PF and fibrin gels was kept constant at 7 mg/ml, roughly double the concentration of fibrinogen found in native blood clots^29^. The contribution of the synthetic component in the PF substrate was used to increase the cross-linking density and sterically mask the fibrinogen, thereby interfering with cell adhesion and invasion into the substrate. Increasing amounts of PEG in the PF formulations were able to heighten the masking effects and cause more pronounced alterations of BM-MSCs seeded on these substrates. For example, BM-MSCs that were seeded on fibrin displayed a polarized, spindle-shape morphology that is consistent with fibroblastic phenotype, whereas those seeded on PF formulations formed aggregates and nodules whose size correlated somewhat with the PEG concentration. One parameter that confounds the PEG masking effect is the stiffness of the biomaterial, which we and others have also demonstrated to correlate well with cell morphogenesis and differentiation^26^,^27^,^30^,^31^. Stiffness in the PF hydrogels is governed by the amount of cross-linker, or the concentration of PEG-DA, which also increases the steric shielding of the fibrinogen^32^, thereby complicating our efforts to differentiate between the response to reduced cell adhesion, cell invasion and substrate stiffness. Therefore, it is possible for two formulations of PF to display similar aggregate surface areas, yet vastly different expression of chondrogenic markers. The PF3 and PF5 are a case-in-point; the stiffness of PF5 being more than 6-fold higher than that of PF3 could be a plausible explanation for this observation.

Based on our results, we were able to deduce that at least one of the substrate’s properties—the permeability towards cells—is not at play and unlikely to contribute to the observed morphogenetic trends. Even though restricting the cells to the surface of the gel vis-à-vis a reduced permeability could minimize cell-substrate interactions and maximize cell-cell interactions, the BM-MSC aggregation on PF hydrogels is probably less affected by this factor because we found invasion to be quite similar across all PF formulations tested (data not shown). Accordingly, this finding suggests substrate permeability was not dominating the observed PEG-dependent morphogenic response. Instead, we hypothesize that the main factor that influences cell aggregation is the steric hindrance of the cell adhesion motifs present on the fibrinogen due to PEGylation. This steric interference with cell adhesion can possibly favour cell-cell interactions rather than cell-substrate adhesion, leading to more pronounced aggregation in a PEG-dependent manner. Due to cell clustering on the PF hydrogels, we utilized the metabolic dye Alamar blue rather than relying on cell counting assays. This approach has been used previously to follow retained cells and cell clusters on substrates^33^,^34^. Indeed, the BM-MSC adhesion to PF substrates early after seeding was found to be significantly reduced when compared to fibrin (Fig. 3b). Moreover, there was an inverse correlation between PEG content and cell retention. Interestingly, by 18 hours post seeding, this correlation was diminished and all PF formulations bound cells similarly to TCPS. We speculate that under conditions that favour reduced cell adhesion, the cells are more mobile on the substrate surface and thus have a higher chance of association with neighbouring cells. As the concentration of PEG increases further, we speculate that the substrate becomes less adhesive and more difficult for cells to navigate, limiting further aggregations. Similar non-linear response patterns were also observed in relation to the matrix stiffness in our system. A recent study by Tam *et al.* showed a connection between BM-MSC adhesion and substrate stiffness^34^. They demonstrated that PCL films with lower stiffness values bound cells more efficiently than stiffer PCL films. The current study also points to a similar relationship between stiffness and cell adhesion. In this regard, we cannot rule out that higher PEG content in the PF hydrogels had a negative role on cell adhesion not only through steric hindrance but also through increased stiffness of the resulting hydrogels. Another study utilizing 3D hyaluronic acid-tyramine hydrogels encapsulating BM-MSCs showed that the highest aggregation and chondrogenic phenotypes were found using a formulation that is approximately 3-fold softer than the PF3 formulation, whereas other formulations much closer in stiffness to PF3 had the lowest values of these two parameters^35^. It is likely that the discrepancy between this study and the current one is related not only to the 2D vs. 3D configurations, but also to the polymeric and biological components of the two systems. Ultimately, more studies are required to ascertain which of these matrix properties dominates the cell aggregation response in this system^31^.

Cell aggregation, round morphology and reduced cell adhesion were previously implicated as being pro-chondrogenic factors^36^,^37^,^38^,^39^. For example, Eyckmans *et al.* showed that reduced cell spreading yielded better chondrogenic differentiation of human BM-MSCs^40^. Guo *et al.* reported that MSCs cultured on the PAAm- and PEG-modified surfaces expressed high levels of cartilaginous genes compared to control surfaces^41^. They conclude that adhesion, proliferation, and differentiation of the MSCs could be controlled by surface chemistry. In Glennon-Alty *et al.*, modified polyacrylate surfaces were shown to induce chondrogenesis of BM-MSCs prior to their aggregation but that aggregation and condensation of the cells appear to be required for the progression of chondrogenesis on these surfaces^42^. Consequently, they concluded that reduced cell adhesion did not play a role in the chondrogenesis on the PAAm- and PEG-modified surfaces.

We set out to test whether the cell aggregation observed on the various PF surfaces is likewise manifested in enhanced chondrogenic differentiation of seeded BM-MSCs. Interestingly, PF3, a cartilage repair hydrogel that is currently under clinical evaluation for treating focal cartilage injuries (under the trade name GelrinC), was found to be optimal among all tested formulations in supporting chondrogenesis as indicated by the highest collagen II and aggrecan levels (Fig. 4) and the highest collagen-II/collagen-I ratios (Fig. 5). Consequently, the collagen-II/collagen-I ratio represents a chondrogenic index of differentiation^43^, and though usually based on mRNA data, use of this ratio with protein level expression has been documented^44^. Moreover, while formulations with zero PEG (fibrin) or low concentration of PEG (PF1-2) were not as pro-chondrogenic, those with very high concentration of PEG tended to lead to higher expression of collagen-I and collagen-X, both markers of scar and hypertrophic cartilage, respectively. Notably, this study did not include analysis of BM-MSC cultures on pure PEG hydrogels. Pure PEG was shown to be pro-chondrogenic towards encapsulated cells^45^. Similarly, pure alginate was superior to pure fibrin in supporting BM-MSC chondrogenesis in 3D^19^. However, these pure synthetic hydrogels are not suitable to serve as 2D substrates due to their very low cell adhesive properties. Moreover, implants based on pure PEG are prone to expulsion *in-vivo* due to poor integration^46^,^47^.

Although in this study we are able to correlate between cell-cell interactions that occurred on the PF formulations (measured indirectly by aggregate surface areas) and the characteristic hyaline-like phenotype markers, we did not evaluate markers associated with early events in chondrogenesis. In particular, early chondrogenesis can be assessed by evaluating gene expression of cell interaction molecules such as integrins. Winter *et al.* evaluated the gene expression of BMSC for cell interaction molecules and found that many important genes are upregulated and down-regulated as a direct result of chondrogenesis^48^. Future studies with the PF formulations that use gene arrays to document the expression profiles of cell interaction molecules will help to gain a better understanding of the mechanisms by which the PF3 drives chondrocytes towards their characteristic hyaline-like phenotype.

## Conclusion

The current study shows that fibrin, a natural component in healing and tissue repair of cartilage can be altered to display more chondrogenic properties *in-vitro* using simple polymer modifications. Shielding fibrin’s adhesion domains and increasing stiffness of the material are likely contributing factors that ultimately cause BM-MSCs to aggregate, form nodules, and display a more chondrogenic phenotype in 2D culture. This insight is important in light of the sub-optimal clinical outcomes of marrow stimulation procedures (i.e., microfracture) that solely rely on mesenchymal stem cell-rich fibrin clot and which ultimately result in fibrocartilage. Despite the difficulties in identifying the precise mechanism responsible for the observed aggregation and chondrogenic responses, the current study is one of the first to establish some good correlation between hydrogel properties, including stiffness and adhesiveness, and BM-MSC aggregation in 2D. It also illustrates the importance of careful formulation and a modular approach when designing biosynthetic materials that could ultimately be optimized for cartilage repair applications.

## Additional Information

**How to cite this article**: Goldshmid, R. *et al.* Steric Interference of Adhesion Supports *In-Vitro* Chondrogenesis of Mesenchymal Stem Cells on Hydrogels for Cartilage Repair. *Sci. Rep.* **5**, 12607; doi: 10.1038/srep12607 (2015).

## Figures and Tables

**Table 1:** Composition and storage shear modulus (G′) values of tested formulations.

Formulation	PEG-DA(mg/ml)	Fibrinogen(mg/ml)	PEG: Fibrinogenmolar ratio	Storage shear modulus(G′) (Pa)
Fibrin	—	7	0:1	118 ± 7
PF1	17	7	48:1	316 ± 9
PF2	30	7	80:1	1,611 ± 96
PF3	47	7	120:1	5,128 ± 275
PF4	97	7	248:1	15,160 ± 298
PF5	147	7	375:1	33,173 ± 825

Fibrinogen and PEG-DA concentrations were determined using Kjeldahl nitrogen determination and gravimetric methods, respectively; G′ values were determined using time sweep rheological measurements. The table shows average values of G′ and standard deviations based on n = 3 for each formulation.
